# In trust we trust: The impact of trust in government on excess mortality during the COVID-19 pandemic

**DOI:** 10.1177/09520767211058003

**Published:** 2022-04

**Authors:** Bishoy Louis Zaki, Francesco Nicoli, Ellen Wayenberg, Bram Verschuere

**Affiliations:** Department of Public Governance and Management, Faculty of Economics and Business Administration, 26656 University of Ghent, Belgium; Department of Public Governance and Management, Faculty of Economics and Business Administration, University of Ghent, Belgium Department of Economics, 1234 University of Amsterdam, the Netherlands; Department of Public Governance and Management, Faculty of Economics and Business Administration, 86796 University of Ghent, Belgium; Department of Public Governance and Management, Faculty of Economics and Business Administration, 86796 University of Ghent, Belgium

**Keywords:** COVID-19, comparative public policy, policy learning, trust, crisis response

## Abstract

The COVID-19 pandemic has brought forward myriad challenges to public policy, central of which is understanding the different contextual factors that can influence the effectiveness of policy responses across different systems. In this article, we explore how trust in government can influence the ability of COVID-19 policy responses to curb excess mortality during the pandemic. Our findings indicate that stringent policy responses play a central role in curbing excess mortality. They also indicate that such relationship is not only influenced by systematic and structural factors, but also by citizens’ trust in government. We leverage our findings to propose a set of recommendations for policymakers on how to enhance crisis policymaking and strengthen the designs of the widely used underlying policy learning processes.

## Introduction

How governments respond to contain the spread of Covid-19, and the consequences of those responses, pose challenging questions for both the research and practice of public policy and administration. As a global exogenous shock, the Covid-19 crisis provides opportunities for learning by comparing a wide range of responses across different contexts (e.g., Capano et al., 2020; Dunlop and Radaelli, 2020; Rose, 1991). The outcomes of such learning hold significant theoretical and practical relevance, particularly given the societal, health, and economic implications of Covid-19 policy responses. This emphasizes the need for furthering the research agenda on COVID-19 crisis responses while maintaining relevance, rigor, and an eye on implications for the practice of policymaking (Dunlop et al., 2020; Migone, 2020; Moon, 2020).

A central component of such research agenda is the intense debate over the stringency of containment measures such as lockdowns, quarantine rules, suspension of educational activities and public assembly. Public reactions to lockdowns and other policy measures have varied across different countries, ranging from instances where decisive government action enhanced approval ratings to others where similar actions were viewed as setbacks to civil liberties or the economy (e.g., Bekker et al., 2020; Popelier, 2020). The latter reaction became more salient as the outbreak gradually became less visible to the public. While most governments initially focused on the immediate public health demands; one and a half year into the pandemic, the social and economic effects ensuant to such containment measures are undergoing increasing public scrutiny. Given the high multi-dimensional price tag on containment measures, achieving low mortality as an outcome becomes key. Yet policy responses have largely varied across countries either in stringency or timing. Perceptions of the pandemic, politico-administrative traditions, and issue framings have influenced public administrations and politicians’ policy preferences. This yielded a range of approaches to the debate on public health and the economy from directive to communitarian to hollow and coping states (Van der Voet, 2021). In some countries, such as Sweden and South-Korea, governments initially focused on maintaining the economy afloat, which in turn directed efforts to protecting the most vulnerable and avoiding large-scale lockdowns whenever possible (e.g., Lee et al., 2020; Pierre, 2020). Other countries such as China, France or Italy opted for stringent early-on lockdowns (Capano et al., 2020), with some countries (particularly in Europe), shifting from the softer to the harder approaches as they observed deficiencies in initial mitigation (Jae Moon, 2020: 655). Here, the dynamism and variations in COVID-19 policy responses emphasized three main issues. *First*, the nature of COVID-19 as a technically complex, highly ambiguous emerging problem meant that scientific consensus on a single optimal course of action can be difficult to obtain (Van Dooren and Noordegraaf, 2020). *Second*, COVID-19 policy responses such as non-pharmaceutical interventions (NPIs) including updated hygienic standards, social distancing, and lockdowns are socially and behaviorally moderated (Cao et al., 2020). Thus, their effectiveness is non-universal across various policymaking contexts (national and sub-national). *Third*, the viable courses of action are contingent on the systemic, economic, socioeconomic, and politico-administrative contexts of different countries. This emphasizes the need for a deeper understanding of the contextual configurations under which COVID-19 policy responses can yield their aspired outcomes. Emerging research has attempted to show the temporal impact of early versus late action (e.g., Migone, 2020). However, more than one and a half years on, comparative research leveraging rich COVID-19 data aiming to explore such contextual configurations remains scarce.

In this article, we focus on the notion of “trust” as a potential moderator for the impact of NPI-driven policy responses on excess mortality by drawing on data from a set of 27 countries over a 52-week period starting the beginning of the pandemic. Some recent studies have illuminated aspects of individual behaviors that influence compliance to COVID-19 policies (see for instance Chernozhukov et al., 2020). In this article, we complement such studies by exploring, at the aggregate level, the moderating effect of trust. Here, we circumvent issues such as the lack of repeated observations at individual level, and the difficulty of establishing a direct link between individual behavior and aggregate mortality.

Our choice of variables has three main motivations pertinent to plausibility of impact, availability of robust measurements, and measurement equivalence (George et al., 2020). First, NPIs are established to be socially and behaviorally moderated. Here, burgeoning COVID-19 literature is rife with indications of how trust plausibly influences compliance (e.g., Toshkov et al., 2020; Devine et al., 2021). This is in addition to pre-pandemic literature offering similar indications (e.g., Widaningrum, 2017). Second, there are robust measurement schemes for trust and excess mortality, chief of which is the Eurobarometer trust data and official equivalized excess mortality information (European Commission, 2021a, 2021b). Third, the standardized availability of data for a number of countries allows for cross-country comparisons while maintaining measurement equivalence.

Our exploratory investigation into how trust in national governments moderates the effect of containment measures (represented by the stringency of policy responses) on the ultimate indicator of public health during pandemics (represented by excess mortality) is guided by two central research questions:

(1) Is there an effect of containment measures on the number of Covid-19-related excess mortality, and if so, to what extent?
(2) To what extent does trust in government moderate the effectiveness of policies on excess mortality?

The contribution of this article is threefold. Theoretically, we further elucidate the relationship between trust in government and compliance during fast-burning wicked crises while accounting for different potentially confounding issues (e.g. healthcare capacity, population density, etc.). Empirically, we create a novel account of a relatively new phenomenon across 27 nations, that is, the impact of trust on policy outcomes during fast-burning crises. Furthermore, as all the nations in our dataset have established COVID-19 scientific committees as a part of an ongoing process of epistemic policy learning (i.e., learning from experts), our findings call for expanding the horizon on the relevant expertise to be called into the policy learning process underlying the crisis policy formulation. Our results also present policymakers with key insights as to different viable approaches concerning policy stringency under different configurations of trust. This assists in reducing the aforementioned multi-dimensional price tag of crisis policy responses by converging on functional modes of policy responses within specific contextual conditions.

## Public policy, guidelines, and trust during the pandemic

In this section, we draw on literature from public policy, policy learning and emerging COVID-19 public administration to elucidate theoretical pathways between trust (from here on to indicate “trust in government”) and policy outcomes (expressed in excess mortality during the pandemic).

First, as we proceed to explore how can levels of trust influence excess mortality within a pandemic, we must first start with the underpinnings of policymaking within such context and their foundational linkages to trust. As a wicked crisis of technical complexity, knowledge gaps and high uncertainty, literature shows that formulating policy responses to the COVID-19 pandemic largely relies on a process of epistemic policy learning. That is, learning from groups of experts with authoritative claims to subject matter knowledge (Dunlop and Radaelli, 2013; Zaki and Wayenberg, 2020). In such conditions, a limited group of experts (commissioned, certified, and consulted by national governments) provide critical knowledge that underpins and drives policy action. Here, a clear link can be observed between the existing levels of trust in governments and public trust in their COVID-19 policy responses (naturally inclusive of guidelines resulting from government commissioned expertise).

The relationship between such trust and compliance with government regulations has been previously studied and recently emphasized (e.g., Devine et al., 2021). For example, Harring and Jagers (2013) found that political trust significantly affects people’s attitudes towards an increased tax on carbon dioxide. Likewise, and inversely, Widaningrum (2017) showed that non-compliant behaviors in Indonesia regarding the law on traffic and road transport, and the local regulation on street vendor management highlighted a form of low-trust in government. As such, they measured the trust—effect on a case-specific level. Furthermore, a positive relationship between trust in government and policy compliance has been repeatedly pointed out at different levels (e.g., see Ejelöv and Nilsson 2020; Konisky et al., 2008). However, while trust in government in fast-burning wicked crises can be critical for enhanced crisis response, this does not necessarily imply that such trust is normatively or inherently positive in other contexts. Absolute forms of trust and the lack of healthy skepticism can mask governmental organizations’ underperformance or corruption (e.g., see: (Alon–Barkat, 2019)).

In the case of COVID-19 policy responses, we see burgeoning evidence as to the plausibility of such relationship (e.g., Bargain & Aminjonov 2020; Devine et al., 2021; Saechang et al., 2021; Toshkov et al., 2020). For instance, Bargain & Aminjonov (2020) found that in regions where trust in policymakers is high, compliance to containment measures is higher (measured as decreased non-essential mobility). Hence, given the behavioral contingency of compliance to COVID-19 policy responses, and emerging indications from COVID-19 research, the relationship between trust in government and the acceptance of such policies and guidelines is theoretically and empirically plausible. This renders trust a potential key determinant of government action effectiveness (particularly when focused on such NPIs as key policy instruments). Citizens might be keener to follow guidance and regulations when trust is higher rather than lower. So, ceteris paribus, the effectiveness of given measures is expected to be higher when trust in government is high rather than low. Importantly, even though our baseline hypothesis for trust (H2) indicates that there will be a positive interaction between trust and containment measures, it is worth mentioning that the effect might also play in the opposite direction. It might be that in case of high levels of trust in the competence of government and public administrations, people may underestimate the risks and thus be less inclined to take individual responsibility making containment measures less effective (see, e.g., Devine et al., 2021; Dryhurst et al., 2020; Mei Ling Wong and Jensen, 2020).

**Figure 1.**
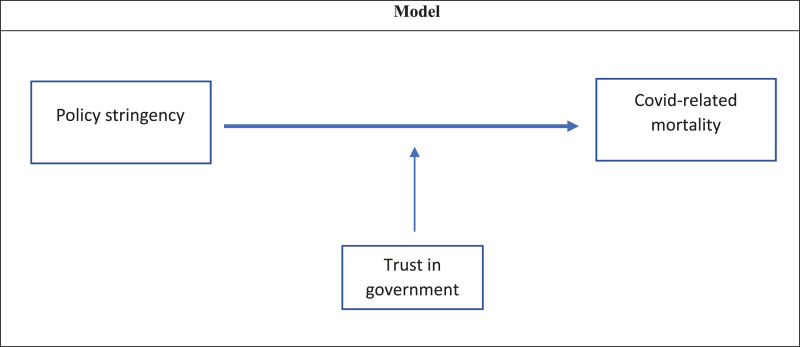
Model.

We develop the following model with independent, dependent and moderator variables, and related hypotheses (see Figure 1). We expect a direct and negative effect of stringent containment measures on Covid-19-related mortality, since after a certain amount of time-*stringent measures lead to decreased contagions, hence, lower mortality (H1)*. During the first year of the pandemic, we increasingly see evidence showing a negative relation between government interventions through containment policies and the spread of the virus (Deb et al., 2020; Dehning et al., 2020; Zongo et al., 2020), so it is plausible to infer that this will also affect mortality. In this article, we will focus on testing the interaction between policy and trust in government, on the desired outcome of that policy (in our case: low mortality). We expect that *the higher the trust is in government, the higher the effect of containment measures on Covid-19-related mortality (H2).* Given extant literature, it is also plausible to expect that people’s opinion of the government and public institutions affects how they comply to containment measures, and therefore—ultimately—affects the effectiveness of the measures themselves (Devine et al., 2021).

In the same vein, the relationship between policy (stringent containment) and outcome (mortality) may be affected by several other contextual variables, like population density, mobility, healthcare system capacity, features of the economy (e.g., opportunities to work from home, or scale of informal economy), national culture, even average temperatures in the region at hand (Cao et al., 2020; David and Pienknagura 2020; Deb et al., 2020). In our analysis, we opt to control for two important variables given the nature of COVID-19 as a highly transmissible contagion: population density and healthcare system capacity. First, in densely populated regions containment measures usually need to be more stringent in order to yield the same effect compared to less densely populated areas. The reasoning is simple: we expect a direct effect of population density on Covid-19-related mortality given that the viral reproduction rate is lower when the population is less concentrated (Rocklöv and Sjödin, 2020). We expect this to be the case because, ceteris paribus, when population density is high, the marginal gain from more stringent lockdowns is higher, than when the population density is low. Second, we can expect that Covid-19-related mortality is also a function of the healthcare system capacity in a region/country. In countries with a robust and resilient healthcare system and where the capacity has not been exhausted at any point during the pandemic, the effect of containment measures on the mortality may be smaller, hence, the need for stringent measures may be less compelling. Healthcare quality indicators may be expected to have a strong effect of their own, so lockdowns would be less necessary and less effective when the healthcare system is very robust.

## Data and methods

Our empirical scope is 27 countries from continental Europe with data from 52 weeks over the year 2020.^1^ This choice is made based on practical considerations including availability of robust data and measurement equivalence as earlier illustrated in our introduction section (See: George et al., 2020). For an overview of our cases and data, see Annex 1.

### Dependent variable

Choosing the dependent variable for this study is a delicate endeavor. Several dependent variables exist, and many have been used in recent Covid-19 literature to assess the effectiveness of policy measures. Intuitively, the number of infections comes to mind as one of the main variables used in earlier reports. While the underlying variable certainly captures the sought outcome of lockdowns (which is not to directly reduce, say, mortality rates, but rather to contain the spread of the virus), it suffers from a variety of measurements problems. Data is dependent on national reporting standards and case definitions (which may differ) and is especially sensitive to varying testing capacities and strategies. As a consequence, even though conceptually this would appear to be the most directly relevant variable to assess the effectiveness of lockdowns, in practice available data diminishes its significance in comparison to alternatives. The estimated reproduction rate (R0) suffers from similar concerns.

A second approach looks instead at the number of Covid-19-related fatalities, capturing the most dramatic underlying material effect of the pandemic. Lockdown measures do not directly target this variable but reducing the number of fatalities due to the viral outbreak remains, of course, the ultimate goal of any policy response, so this constitutes a suitable variable in principle. This ultimate goal however was approached varyingly by different governments given contextual configurations and policy priorities as previously established. While some have pursued harder early-on lockdowns, others have opted to keep their economies afloat by focusing protective measures on vulnerable populations. However, this measure still suffers from similar issues as the number of infections: countries differ substantially in what they count as a “Covid-19-related fatality.” These differences may be due to genuine differences in beliefs regarding what constitutes COVID-19 mortality^2^ but sometimes they may also be due to political legacies and ideological dispositions. Hence, once again, this variable is not highly suited for cross-country comparisons.

A third alternative implies a step beyond simply counting the number of directly recorded Covid-19 deaths and looking at the differentials between the overall country-wide death rates in 2020, and the death rates in previous years. This provides an overall view of how grim a country’s situation during the pandemic is, at the cost of foregoing precision on the specific causes of death (which nevertheless remains a controversial issue). For instance, the death differential may include deaths directly caused by Covid-19 complications, or by the interaction between Covid-19 infections and pre-existing conditions, and deaths caused by other Covid-19-related problems (deaths resulting from decanting hospitals, reduced access to treatment, care facilities, or medical “professionals” due to Covid-19-related resource exhaustion). The variable also embeds *decreases* in the death rate which may be attributed to lockdown measures, but not Covid-19-related, for instance, a reduction in traffic fatalities, and the reduction in circulation of other pathogens (like seasonal flu or influenza). For these reasons, the death rate differential can be a more suitable choice of variable for cross-country comparisons despite being less nuanced from a clinical point of view. Nonetheless, as this article aims to explore varying outcomes in relation to different policy alternatives as well as the impact of trust, being able to delineate causes of death at utmost accuracy takes a second seat to being able to conduct robust cross-country comparisons. Hence, we opt for the death rate differential as our main dependent variable. Death rate differentials are sourced from the European Union’s normalized excess mortality data. This data is collected for the 52 weeks of 2020 for 27 European countries.

### Independent variables

This study looks, specifically, at the potential influence of containment measures on reducing mortality during the COVID-19 pandemic. Our main independent variable of interest is the stringency of national policy responses to COVID-19, mainly in terms of containment and closure policies. To capture this variable, we take the Oxford policy Stringency Index (OSI) (University of Oxford, 2020). The index consolidates eight stringency indicators for closure and containment policies such as school closures, restrictions of mobility, and cancellation of public events. As a composite index, the OSI must be interpreted carefully: since the initial components do not necessarily compensate for each other, the construction of the index in itself reflects certain choices made by the original researchers. Furthermore, some components of the index may be more prone to be affected by public trust than others (Goldfinch et al., 2021). Therefore, the advantage of adopting an aggregate view on policy stringency needs to be traded off against the difficulty of establishing (by the mere use of the aggregate index) which alternative measures are the most effectives. Yet, the OSI provides a convincing and widely acknowledged aggregate indicator fit for the purpose of this article, which is not to identify the most effective policy options available to policymakers but to assess the moderating effects of trust.

Similar to the excess mortality rate, the Stringency Index is computed weekly for all countries in our sample. It is important to note, however, that the simultaneous relationship between the OSI and death rate differential is *reversed:* countries where the pandemic is having a stronger effect will in turn start adopting more drastic measures. Furthermore, when policy interventions are announced, time is needed for them to be adequately disseminated, for citizen behavioral adjustments to take place, viral reproduction rates to decrease and thus inducing observable effects on mortality. To detect the effect *from* changes in stringency *to* reductions in the death rate, thus, we need instead to use a lag of the variable (i.e., today’s death rate differential is impacted by yesterday’s stringency). To identify from which point onwards, we start seeing the effects (if any) of stringency on death rates, we first recur to simple correlations.

Table 1 reports the simple correlation between the OSI lags and the death rate differential. As shown, the relationship between the lagged OSI and the death rate differential turns negative from the fourth week backwards, which suggests that containment measures need a minimum of 4 weeks to start being effective.

**Table 1.** Correlations between OSI lags and death rate differentials.


OSI lag	Correlation with death rate differential (excess mortality)
Stringency (1 week lag)	0.28
Stringency (2 weeks lag)	0.21
Stringency (3 weeks lag)	0.11
Stringency (4 weeks lag)	0.00
Stringency (5 weeks lag)	−0.08
Stringency (6 weeks lag)	−0.14
Stringency (7 weeks lag)	−0.17
Stringency (8 weeks lag)	−0.18
Stringency (9 weeks lag)	−0.17
Stringency (10 weeks lag)	−0.16

To avoid endogeneity concerns by means of reverse causality, this analysis will therefore use OSI lags of a minimum of 4 weeks or above.

### Moderating variables

We look at several measures of *trust,* capturing the country average scores for trust in *public administration, national governments,* and *local authorities.* These are relatively correlated items which capture the extent to which citizens believe the institutions within their countries can be trusted. Data is aggregated from the Eurobarometer dataset at country-level. An obvious problem encountered in the process is that, even though the most recent Eurobarometer data have been collected during the pandemic, it is not necessarily fully reliable for our purpose. Chiefly because, once again, we could be facing a problem of reverse causality; the capacity of governments and public administrations to deal with the pandemic is deemed to affect the public opinion and trust in government. The only way to consider such variables as exogenous is to source them from the latest opinion poll data collected before the pandemic hits, namely the July 2019 wave. By using trust data collected just before the pandemic, we are able to minimize reverse causality concerns, reducing the potential sources of bias in our estimates. However, in doing so, a new challenge emerges; since these become time-invariant effects, using the pre-pandemic trust values substantively reduces the power of the study. To circumvent this problem, we add a second version of the variable, which is instead time-variant, using the latest Eurobarometer data (93.1). These are then used for every week starting from week 27 of 2020, allowing us to maintain a larger effective dataset.^3^ Both the static and dynamic versions of trust are used in this study.

### Control variables

Intensity of containment measures is certainly not the sole factor influencing the impact of the Covid-19 on the death rate differential. Even though other influencing factors are not within the primary focus of this article, healthcare capacity features (such as the healthcare spending per capita, the number of intensive care beds, and the number of doctors) can certainly influence our dependent variable. Furthermore, structural features of the country (such as the population density or the share of population living in cities) are also expected to have both direct effects on the fatalities, and mediate the effect of the containment measures as such policy outcomes are known to have multiple interactions with micro, macro, and meso level variables (Peters, 2020). For instance, we expect population density to strongly moderate the effect of containment measures. It is also plausible that higher quality of the healthcare system inversely affects the effectiveness of lockdowns-that is, the marginal gain from lockdowns is lower when the healthcare is very efficient. For these purposes, we use three indicators, obtained from the Eurostat, and World Bank data: per capita health expenditure, number of doctors, and number of Intensive Care Bed Units (per 100,000 of the population) (see Annex 1).

### Timeframe and level of observation

Ideally, a study looking at the aggregate effects should aim to have the smallest possible level of aggregation, for instance at regional or even municipal level. This would be efficient both in increasing the units of observation, and—by doing so—introducing more variance in the data and therefore refine the precision of the estimates. Unfortunately, at the time of conducting this analysis, regionally disaggregated data on mortality rate differentials and on stringency index remain scarce. Initially, the data for Switzerland, Norway, and Iceland was considered, however, was later dropped due to the lack of trust data (not collected as part of the Eurobarometer Survey).

As a result, our dataset covers a period of 52 weeks in 2020; yielding a weekly-based, country-level panel of a nominal total of 1396 weeks datapoints across 27 countries. As some variables are not available on all periods in all countries, and because the use of lagged independent variables reduces the actual number of periods available, our models are usually estimated on a reduced dataset ranging between 1140-1280-country-week observations. Because death rate differentials are likely to be correlated over time as the pandemic advances, we construct models inclusive of the lag of the dependent variable to account for time dependency, as discussed in the models’ section.

### Model specifications

The data follows a panel structure, with weekly observations at the country-level. The nature of the dependent variable—highly correlated over time—invites the inclusion, on the right side of the equation, of a lag dependent variable. We are therefore faced with a dynamic panel structure, where the time *t* value of the dependent variable is not only determined by the independent variable, but also by its own level at time *t-1*.^4^

We proceed in two steps. In a first step (Table 2), we estimate a series of baseline models where we keep the right side of the equation to the minimum, to ensure comparability across models. We label these as “A-models.” All A-models regress the excess mortality on its first lag and the fourth lag of the stringency index. Model A1 is a simple OLS with cluster-adjusted Standard Errors. Model A2 is a FE estimator; model A3 is a Random Effects (RE) estimator; model A4 is an AB dynamic panel model, and model A5 is the same as A1 but augmented with controls.^5^ These models allow us to test H1. As shown in the next section, the relationship between the coefficient estimates across these models is very stable, which suggests that our baseline estimations are robust.^6^ Next, we augment Models A1 and A3 (for which it is possible to compute coefficients for time-invariant variables) with various independent variables (models B1–B10). These models (B7–B9 in particular) allow us to test H2.

**Table 2.** Baseline estimates comparison.

	A1 OLS (cluster)	A2 FE	A3 RE	A4 AB	A5 OLS (cluster) controls
Excess mortality (previous week)	0.875***	0.887***	0.875***	0.873***	0.893***
	−0.034	−0.0146	−0.0152	0.015	(0.0148)
OSI (4 weeks lag)	−0.0532***	−0.0492***	−0.0532***	−0.0774***	−0.0492***
	−0.0176	−0.0125	−0.0129	−0.0142	(0.0127)
1000s of doctors					−0.000454
					(0.352)
Health spending per capita					−0.000232
					(0.000149)
Density per square km (1000s of people)					5.468*
					(2.869)
Country dummies (omitted)	Omitted	n/a			
_cons	4.194***	4.087***	4.388***	5.503***	3.984**
	−0.665	−0.624	−0.643	−0.695	(1.650)
N	1336	1336	1336	1308	1240
Groups	28	28	28	28	28
R^2^	0.747		0.725		0.763

*p < 0.1; ** *p* < 0.05; *** *p* < 0.01.

^a^Note: the large R-squared for the two fixed effects models are imputable to both the presence of the fixed effects themselves, and of the lagged dependent variable in the equation.

## Results

Table 2 reports the estimations for models A1–A5. The first column reports baseline estimates of a simple OLS with country fixed effects and clustered standard errors. The second column uses instead a Fixed Effects (FE) panel estimator. The third model is a Random Effects (RE) panel estimator, while the fourth model is an Arellano-Bond (AB) dynamic panel model. All four models offer a very consistent picture, with statistically non-significant differences between the estimated coefficients of the two variables included (the 4-weeks lag of the OSI and the 1-week lag of the dependent variable itself). The proximity of these indicators to each other lends credibility to the strategy of proceeding with the estimation of models inclusive of time-invariant variables, which is not possible in FE and AB models (the latter probably being, otherwise, the most suitable estimator; results from an augmented version of the AB estimator-the Kripfganz and Schwarz (2015)’s xtseqreg estimator-are reported in appendix 3.2.

In general, results from models A1 to A5 are very stable across estimators. This is not only true for the average effects, but also for their marginal dynamics: Table 3 reports the predicted excess mortality at four OSI points of interest (0, i.e., the minimum; 1; about one unit; 33, about one standard deviation; and 90, about the maximum). An increase in one standard deviation of the fourth lag of the OSI leads to a decrease in excess mortality, on average across models, of about 0.87 points, or about 2%. This may seem small to start with, but it is to be considered the effect attributable to the *fourth week,* not over the entire period as indicated in Table 1. As a result of these models, we can preliminarily fail to reject H1: across all models, we find a stable and negative effect of the fourth week’s stringency level on excess mortality. In addition, model A5 shows that the estimate on the effect of the OSI is fundamentally robust even when additional controls are included, supporting H1. We aim, of course, to refine these results with wider models; we therefore turn to the outcomes of models presented in Table 4 to better understand how stringency behaves in association with other variables.

**Table 3.** Predicted levels of excess mortality at chosen OSI (lag 4) values.

	OLS (cluster)	FE	RE	AB
OSI(4) = 0	13.1	13.1	12.9	14.2
OSI(4) = 1	13.1	13.1	12.9	14.1
OSI(4) = 33	11.4	11.4	11.3	11.7
OSI(4) = 100	7.8	7.8	8.0	6.5

**Table 4.** OLS (cluster-robust) estimates on full model & interaction models.

	B1 Full model, no interactions, trust in government, dynamic variable	B2 Full model, no interactions, trust in PA, dynamic variable	B3 Full model, no interactions, trust in local authorities, dynamic variable	B4 Full model, no interactions, trust in government, static variable	B5 Full model, no interactions, trust in PA, static variable	B6 Full model, no interactions, trust in local authorities, static variable	B7 Government trust interaction	B8 Trust in PA interaction	B9 Trust in local public authorities interaction	B10 population density interaction
Excess mortality (previous week)	0.912***	0.913***	0.913***	0.912***	0.912***	0.912***	0.911***	0.913***	0.913***	0.897***
	(0.0231)	(0.0230)	(0.0230)	(0.0228)	(0.0228)	(0.0230)	(0.0233)	(0.0231)	(0.0233)	(0.0311)
OSI (4 weeks lag)	−0.0554***	−0.0557***	−0.0555***	−0.0562***	−0.0562***	−0.0559***	−0.0721	−0.0433	−0.0605	−0.00587
	(0.0168)	(0.0167)	(0.0167)	(0.0168)	(0.0166)	(0.0167)	(0.0503)	(0.0380)	(0.0498)	(0.0199)
Trust in government, PA or public authorities (direct effect)	−0.0239**	−0.0196*	−0.0184*	−0.0303**	−0.0281***	−0.0266**	−0.0435	−0.00881	−0.0225	
	(0.00970)	(0.00955)	(0.00952)	(0.0110)	(0.00944)	(0.00998)	(0.0515)	(0.0346)	(0.0415)	19.12***
Density per square km (1000s of people)	3.676**	3.311**	3.271**	3.911***	3.711**	3.543**	3.632**	3.337**	3.264**	(4.446)
	(1.386)	(1.390)	(1.533)	(1.383)	(1.375)	(1.559)	(1.327)	(1.356)	(1.511)	
OSI#trust (interaction)							0.00	−0.00	0.00	
							(0.00102)	(0.000628)	(0.000767)	
OSI#Density per square km (interaction)										−0.325***
										(0.0768)
_cons	4.698***	4.806***	4.805***	4.911***	5.204***	5.232***	5.482**	4.245**	5.036*	1.470
	(0.905)	(1.020)	(1.011)	(0.968)	(1.039)	(1.038)	(2.494)	(2.046)	(2.628)	(0.910)
*R*^2^	0.791	0.793	0.791	0.791	0.791	0.791	0.791	0.791	0.791	0.764
N	1096									1240

Hence, Table 4 reports the estimates for variants of the first baseline model The first column includes model B1, that is, a variant of A1 augmented with the dynamic version of the trust in government indicator. Model B2 features the dynamic trust in Public Administration, while model B3 includes instead dynamic trust in local public authorities. Models B4–B6 have the same features, but with the static version of the trust variables instead. There are trade-offs when choosing between dynamic and static versions of trust. The static version of trust is measured *prior* to the beginning of the pandemic, and therefore allows us to rule-out reverse causality. However, it only includes one-time period, and therefore it decreases the variance in the dataset and the power of the study. On the other side, using the dynamic version of the trust indicators allows us to maintain a larger effective *n* in the study, but at the cost of possible endogeneity. As shown in Table 4, however, the results of the regression using the dynamic version of the trust variables (models b1–b3) are very close to those using the static version (models b4–b6). This suggests that the bias due to possible reverse causality is relatively contained. On this basis, models b7–b9 provide interaction effects between the dynamic version of the trust indicators and the OSI, and—to conclude—model b10 includes the interaction between population density and the OSI.

**Figure 2.**
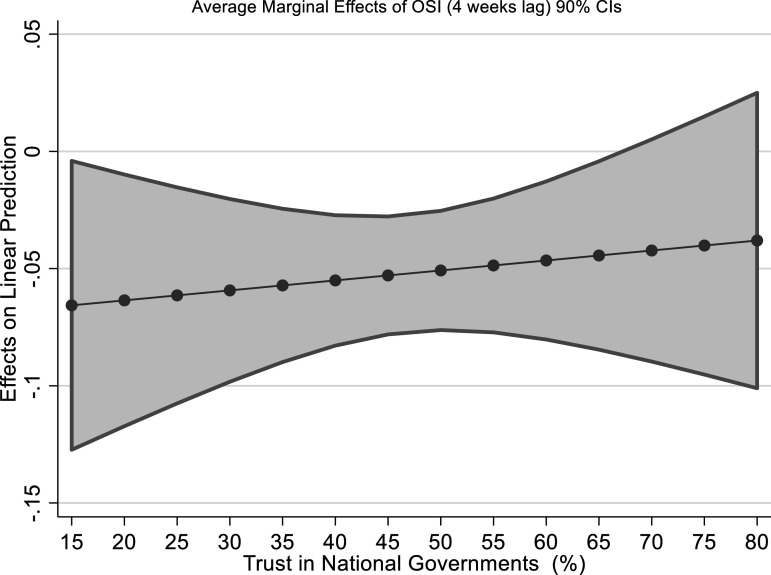
Average Marginal Effects of OSI (fourth lag) on excess mortality at Government trust levels.

Models B1–B6 allow us to test whether trust has a direct effect, in some form, on excess mortality. Across all models, we find that trust in government has a *direct* effect on excess mortality: the higher the trust, the lower the mortality, regardless of the stringency level of containment measures. The effect is substantial, for it is about half the size of the effect of the stringency index. But are stringency and trust mutually reinforcing, or are they substitutes? The *average* interaction effect is of little help because it is not significant, suggesting that, even if trust moderates in some direction the effectiveness of containment measures, the effect is indifferent from zero on average. However, regardless of the statistical significance of the average effect (which ultimately depends on sample size), it is important for our question. To analyze it, we turn at a graphical representation of the marginal effects of the interactions. Models B7–B9 allow us to explore these moderating effects. The marginal effects of these interactions are shown in Figure 2 (for trust in government) go in the direction of rejecting H2: trust in governments has a *positively sloped* effect on the effectiveness of containment measures: containment measures are much more effective when trust in government is low, than when it is high. While this effect is on average non-significant, the overall direction of the interaction is clear. In a way, this is consistent with the information provided in models B1 and B4: trust has an effect on its own on excess mortality, indicating that generally trust affects how people react to government indications regardless of formal containment measures, but—as such—trustful people seem not to take stringent containment measures well. The relationship is replicated in the other models for other types of trust, such as trust in Public Administration or local authorities.

**Figure 3.**
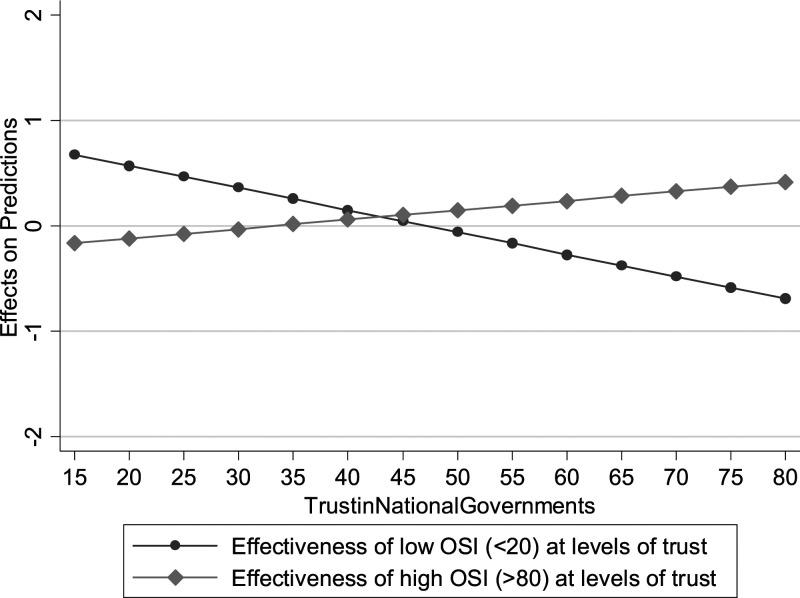
Marginal effects of low and high OSI at levels of trust. Note: these interactions are generally not significant; this is mostly due to the limited number of observations. We display the figures without confidence intervals to highlight the diverging trends in the two subgroups of cases.

**Figure 4.**
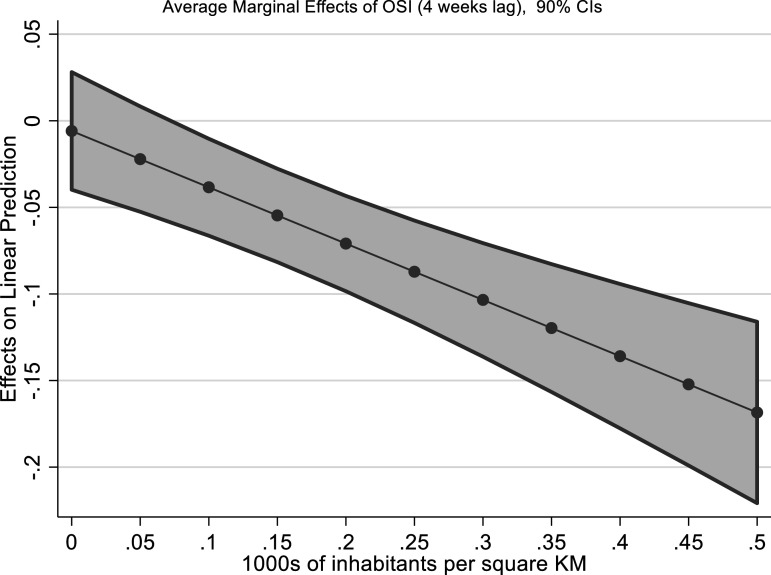
Average Marginal Effects of OSI (fourth lag) on excess mortality at population density.

In sum, the evidence collected is not enough to find support for H2. While these results seem counter-intuitive, one possibility is that trust can function as a proxy for different underlying individual features: for instance, it might be that the countries with very high trust in government are those whose population is also characterized by very strong liberal views when it comes to personal freedom (such as, for instance, the Netherlands and Sweden) where it might be relatively unthinkable to excessively constrain individual rights such as freedom of movement. Hence, trustful individuals might be more likely to respect *non-compulsory recommendations* than relatively less trusting individuals *but less likely to actually comply with extremely stringent regulations that curtail personal freedoms*. We explore this further in Figure 3, which suggests that we might be faced with a composition effect. As shown in Figure 3, when OSI is low, its effectiveness is *increased* by higher trust, while the opposite is true when OSI is high. In other words, societal trust seems to be a good complement to public policy when the measures are not compulsory (and therefore trust in institutions is essential for these policies to work) but seems detrimental when the level of OSI is very high. Finally, in model B10 we look at the interaction between population density and stringency measures. The interaction is strongly negative and statistically significant at 5% level; suggesting that population density is a key moderator of the effectiveness of lockdowns: the higher the population density, the more effective the lockdowns (Figure 4).

## Discussion and conclusions

### Main observations

According to one of the best-known, short, and simple definitions, public policy is “anything a government chooses to do or not to do” (Dye, 1972: 2). That choice-element clearly emerges in case of COVID-19 policymaking. After all, governments could opt for a quite demanding or a rather soft strategy vis-à-vis their citizens to limit the virus’ impact. Hence the varying level of stringency observed in our comparative analysis. Of course, locking down schools, firms, etc., (or in other words: citizens’ “life”) is all but easy from a (democratic) governance perspective. In this article, we analyzed to what extent such containment policies contribute to one of the governments’ ultimate goals within the COVID-19 crisis, saving lives. We also analyzed whether some other factors play a role in moderating or modulating such effectiveness. Our results provide insights on the effectiveness of stringent measures (in terms of curbing excess mortality) and on some of the underlying conditions that make these measures more or less successful. Here, we provide three main observations:

*First*, We find evidence of the overall effectiveness of stringent measures. These are effective in decreasing the impact of the virus in terms of fatalities, all other things being equal. Importantly, also trust in government matters on its own: trust has a direct and negative effect of mortality: ceteris paribus, higher trust societies experience lower excess mortality. Even though such effect should not be interpreted causally, these results suggest that societies characterized by higher trust are also characterized by lower excess mortality; our data however does not allow us to conclude whether such direct effect is causal in nature, or whether it simply captures other unobserved factors that correlate with trust.

*Second*, the effectiveness of such measures is conditional to a series of underlying factors, these include:

• Duration: simple analysis shows that timing matters, in our case it showed that at the very least 4 weeks are required before net effects on mortality become evident. This does not mean that there are no short-term benefits, but the net gains become apparent only after a sufficient period has elapsed.
• Population density: countries with higher population density are likely to be more sensitive to lockdowns, while countries with sparse and highly distributed populations are likely to reap relatively lower benefits from lockdowns.
• Trust interactions: we find no significant evidence *on average* regarding the moderating effect of stronger lockdowns might erode trust in government measures. However, marginal analysis of trust interactions shows some weak indication that, in general, strong lockdowns are slightly less effective in higher trust societies, while soft lockdowns seem to work relatively better in high trust societies than in low-trust ones. Our preliminary data does not allow for conclusive results in this regard and must be treated as purely indicative of a trend.

*Third*, in general, such high societal trust has a direct effect on excess mortality. Even though such effect should not be interpreted causally, this may mean that citizens of high trust societies could be more inclined to respect non-compulsory guidance from their governments. Conversely, low-trust societies seem to require lockdowns more (which naturally comes at a more enforced form), but-as explained above-they are also better to deal with them once they are in place. In other words, there is a degree of replacement between trust and lockdowns; high trust societies might achieve the same results of partial lockdowns with less intensive measures, although this effect diminishes as lockdowns grow stronger.

While statistical analysis makes it somewhat impractical to isolate the sources of variation on a country-level basis, empirical observations can provide further explanatory insights that point in the same direction. Examples such as from Denmark (a country with one of the highest levels of trust in national government, standing at 77%) shows significantly less excess mortality for less stringency. While there are other factors that can moderate such outcomes (e.g., population density and healthcare system capacity), the statistical analysis we conducted has enabled us to control for those factors and isolate the most influential of them.

*Finally,* one important note. We originally expected trust to have no direct effect on mortality but moderating the effectiveness of lockdowns; we expected lockdowns to be *always* more effective in higher trust societies. Instead, we do find a direct and significant effect of trust on mortality, and sparse evidence of a moderating effect on stringency. Furthermore, the direction of this effect counters our expectations, lockdowns seem less effective in higher trust societies. However, given that trust has a strong direct effect, it is also possible that interaction effects should rather be interpreted in the other way around, that is, stringency moderates the effect of trust. If so, then our results show that the *direct* effect of trust on mortality decreases as lockdowns become more stringent—either because these factors tend to substitute for each other, or because stronger lockdowns might erode trust in government measures. The limited data available does not allow us to identify the underlying mechanism with precision, but only to show that both trust and stringency have direct, independent effects, and that the effectiveness of both decreases as the other grows larger.

### Implications for policy research and practice

Next to our empirical observations derived from the case we have studied, there are significant implications for policymaking as a practice, particularly within wicked contexts (such as fighting pandemics).

*First*, the need for context sensitive policy designs is emphasized. All in all, our results suggest that the return on investment for a contextually tailored and carefully crafted containment strategy can be significant, particularly with a focus on influential factors showcased in this article, namely: trust in government, systemic capacities (e.g., healthcare capacity indicators), demographic factors (e.g., population density), and response timeliness. *Second*, mechanisms of policy feedback warrant special attention. Policy measures—stringent or not—feed back into and from society. Hence, policy outcomes in turn affect key aspects of politics and policymaking. As recent survey research shows, government’s success in early fight against Covid-19 increases societal trust (e.g., Beetsma et al., 2020; Edelman 2020). In turn, societal trust can help partially substitute for containment measures, allowing the achievement of similar results with less severe consequences for freedoms and economic sustainability. This is in line with current research in other fields: recent reviews of the policy feedback literature have revealed a variety of ways in which similar transformations take place (e.g., Béland and Schlager 2019; Daugbjerg and Kay 2020).

In the case of the COVID-19 pandemic, signs of such mechanisms feeding back into the design of (future) policy are observable. For example, sub-national governments across Europe are gradually getting more leeway to deal with the crisis on their own jurisdictions. As such, national governments allow more divergence in policymaking, recognizing that COVID-19 measures in urban areas are not automatically best-suited in less densely populated areas or in the countryside. Apart from this approach to national policy and its design, signs are also all around that the public often lacks trust in governmental performance with regard to the containment measures. We observe citizens launching petitions, often via social media, or holding mass protests against government measures. In light of our findings here, the risk that policymakers find themselves in a catch-22 situation is tangible. Trust in government is needed to soften containment measures (the substitution effect we found), but containment measures themselves risk increasing distrust when not carefully crafted.

A *third* consideration is that policymakers need to expand the horizon on expertise within the epistemic policy learning process. As our results show, the effectiveness of policy responses in such crises (particularly when behaviorally moderated and socially embedded) renders policy design and implementation highly complex. Though the crisis can seem overwhelmingly medical in nature, this study shows that societal and demographic factors can have detrimental impacts for medically driven policies. Thus, the technical and societal complexity underpinning such crises calls for expanding the horizons on relevant expertise through a multitude of inter and intradisciplinary resources (Zaki and Wayenberg, 2020). As such the adequate identification of relevant expertise allows for enhanced situational synthesis (Donnelly, et al., 2018). Hence, allowing for a wide range of expert-driven tools to engage with the key determinants of policy effectiveness (such as trust in our case). Such tools can include the use of crafted policy narratives aimed at maintaining and enhancing trust (e.g., Jones and McBeth, 2010; Mintrom & O’Connor, 2020), expert staging of science (Van Dooren and Noordegraaf, 2020), or maintaining trust through a clear delineation of limitations and expectations to the public (Zaki and Wayenberg, 2020). Furthermore, as COVID-19 policies cut across a wide range of sectorial boundaries on the ground, the inclusion of local governments, civil service experts, and practitioners can yield critical insights to formulating effective and efficient policy interventions (Zaki and George, 2021). This emphasizes the need for the underlying policy learning processes (as main drivers of policy responses during such crises) to closely interact with various elements of the crisis context.

*Fourth,* our results show that dimensions of trust in government can mean the difference between life and death during such crises. Thus, systematic longer-term investments in strengthening citizen’s trust in public administration should be considered. This can be through intensive investments in recruiting highly competent civil servants (Wooldridge & Micklethwait, 2020). This is particularly as the civil service’s competence is highly entwined with institutional images, public perception, and citizens’ trust in government as a whole (e.g., Houston et al., 2016). As such, an era of wicked crises, dynamic complex contexts, and accordingly evolving governance paradigms calls for recruitment of civil service cadres with the adequate and relevant cognitive and personal capacities (Kruyen & Van Genugten, 2020).

### Limitations

Our results should be interpreted with some caution given existing limitations of available data. To start with, the number of countries reporting sufficient information upon which estimations can be performed is limited, and these missing observations are potentially non-random. Second, we are forced—by data availability—to take the country level as the level observation, but this constitutes a large aggregator; important regional differences are present within countries, and a country-level estimation is not necessarily best suited to fully grasp the effectiveness of containment measures. In this regard, we consider as essential for future research and to better base future policy responses in data that governments release relevant information disaggregated at least at NUTS-1 level. In general, the more disaggregated the data, the better. This would also allow a much more precise (and therefore, policy-valuable) estimate of the effects of containment measures and containment moderators. Third, the OSI index, as an aggregate index, allows us to look at the overall effects of lockdowns, without however discriminating between individual more or less effective policies. Furthermore, its underlying assumption is that its constituent elements can somehow compensate for each other in achieving a certain score. In a pandemic where different policy mixes and policy sequences have been deployed with different results, this assumption may not necessarily hold.

Furthermore, the models presented here rely on a combination of fixed effects, use of lagged information, and use of pre-pandemic data to approximate causal reasoning. While there is some merit in each of these choices, together they do not constitute a sufficient setup allowing for causal inference, and our results need to be interpreted as exploratory. As data on trust changes and on buildup of medical capacity are released, better estimates and better modelling strategies will become available. Last but not least, while the model utilized in this article offers significant and robust explanatory power, a standardized index for policy interventions’ degree of enforcement that enables statistical cross-country comparisons remains to be developed. Furthermore, the availability of such data will assist expand future research to more global comparative perspective that goes beyond European countries. Thus, we call upon future research to embark on such endeavors.

Additionally, our results should not be interpreted as suggesting that stringent measures are always and invariably the best option to address pandemic events. Importantly, even though we do find a clear effect on mortality rates during the pandemic, our model does not account for potential costs of stringent measures on the economy of EU member-states; an assessment of the latter is required to provide a full picture of benefits and costs of stringent decisions.

These limitations notwithstanding, this article provides strong preliminary evidence on how the effectiveness of containment measures is in part responsive to the cultural and physical infrastructure of a region and offers some interesting insights for policymakers to inspire future actions in the unfortunate but not impossible scenario where stringent containment measures will again been needed in the future.

## Notes

1 Austria, Belgium, Bulgaria, Croatia, Czech Republic, Denmark, Estonia, Finland, France, Germany, Greece, Hungary, Iceland, Italy, Latvia, Lithuania, Luxembourg, Netherlands, Northern Ireland, Poland, Portugal, Scotland, Slovakia, Slovenia, Spain, Sweden, Iceland, Switzerland, and Norway.

2. For instance, it can be debated of whether someone with critical underlying conditions who contracted the virus can be considered as a Covid-19 fatality.

3. Unfortunately, doing so prevents us from excluding reverse causality: it is entirely possible that post-pandemic trust levels are affected by government management of the pandemic itself. However, the static and dynamic trust variables are naturally highly correlated (from 0.97 for local public authorities to 0.92 for national governments) suggesting that trust remained relatively stable over the whole period, and therefore reducing the likelihood and size of possible biases due to two-way causal effects.

4. As suggested by Nickell (1981), when the panel dimension *p* is larger than the time dimension *t,* fixed effect estimators inclusive of lagged dependent variables are biased because they produce a correlation between the error term and the regressors. A class of models, Dynamic Panel Models (DPM) such as the Arellano-Bond (AB) estimator have been developed to deal with this problem. In the case at hand, Nickell bias is less of a concern, since our time dimension substantially larger than our panel dimension (almost twice as large). Even when using a lag of the main independent variable (as described in the previous section), we only lose about three time periods, hence, keeping the bias at minimum. Under these conditions, a fixed effects estimator (FE) should be reliable.

5. A version of model A5 with additional controls is presented in annex 3.2.

6. In annex 3.1 we report, as additional robustness checks, the estimates for the same models using a variety of different lags of the main independent variable, the OSI.

7. The latest working paper on the indicator can be found here: https://www.bsg.ox.ac.uk/sites/default/files/2020-05/BSG-WP-2020-032-v6.0.pdf. For methodology of index calculation: https://github.com/OxCGRT/covid-policy-tracker/blob/master/documentation/index_methodology.md

## ANNEX 1. Information on cases and data sources

### Cases

Austria, Belgium, Bulgaria, Croatia, Czech Republic, Denmark, Estonia, Finland, France, Germany, Greece, Hungary, Iceland, Italy, Latvia, Lithuania, Luxembourg, Netherlands, Northern Ireland, Poland, Portugal, Scotland, Slovakia, Slovenia, Spain, and Sweden.

### Stringency Index (independent)

The Oxford COVID-19 Government Response Tracker (OxCGRT) systematically collects information on several different common policy responses that governments have taken to respond to the pandemic on 17 indicators such as school closures and travel restrictions. It now has data from more than 180 countries. The data is also used to inform a “Lockdown rollback checklist” which looks at how closely countries meet four of the six World Health Organization recommendations for relaxing “lockdown.” Indicators are of three data types; Ordinal, Numeric, Text. Data is collected from publicly available sources such as news articles and government press releases and briefings. These are identified via internet searches by a team of over one hundred Oxford University students and staff. OxCGRT records the original source material so that coding can be checked and substantiated.

Source^7^: https://www.bsg.ox.ac.uk/research/research-projects/coronavirus-government-response-tracker

### Excess mortality

“Death” means the permanent disappearance of all evidence of life at any time after life birth has taken place. Eurostat’s recommendation for the definition of time of death is by “date of occurrence," but data by “date of registration” are also accepted. “Excess Mortality” is the rate of additional deaths in a month compared to the average number of deaths in the same month over a baseline period. The higher the value, the more additional deaths have occurred compared to the baseline. A negative value means that fewer deaths occurred in a particular month compared with the baseline period.

Source: https://ec.europa.eu/eurostat/statistics-explained/index.php?title=Excess_mortality_-_statistics

Dataset: https://ourworldindata.org/excess-mortality-covid

Trust Indicators: Eurobarometer data version 91.5 (June–July 2019) and Eurobarometer data version 93.1 (July–August 2020)

https://dbk.gesis.org/dbksearch/sdesc2.asp?no=7649&db=e&doi=10.4232/1.13671

https://search.gesis.org/research_data/ZA7576

Standard English Survey: https://dbk.gesis.org/dbksearch/download.asp?id=66908

Acute Healthcare beds: Eurostat

https://ec.europa.eu/eurostat/statistics-explained/index.php/Healthcare_resource_statistics_-_beds

Number of Physicians: World Bank

https://data.worldbank.org/indicator/SH.MED.PHYS.ZS?name_desc=true

Number of Medical Nurses: World Bank

https://data.worldbank.org/indicator/SH.MED.NUMW.P3

Healthcare spending per capita (EUR): Eurostat

https://ec.europa.eu/eurostat/databrowser/view/HLTH_SHA11_HF__custom_584545/default/table?lang=en

Population

https://ec.europa.eu/eurostat/databrowser/view/demo_pjan/default/table?lang=en

## ANNEX 2. Descriptive statistics

**Table 5.** Descriptive statistics.

Summary statistics					
Variable	Observations	Mean	Std. Dev	Min	Max
OSI	1396	46.8	25	0	96.3
Excess mortality	1396	9.1	21	−29	156.3
Trust in national governments	1240	40.6	16.3	14.7	77.4
Trust in local public authorities	1240	57	14.5	20	80.6
Trust in public administration	1240	53.9	15.9	23	89.6
Number of medical doctors (1000s)	1240	3.8	0.9	2.4	6.4
Health spending (1000$ per capita)	1240	3211	2071	586	8327
Population density (1000 people per km2)	1292	0.132	0.11	0.003	0.5

## ANNEX 3. Additional estimations

**ANNEX 3. Additional estimationsTable 6.** Table 3 with different selected lags of OSI.

	2 weeks lag				7 weeks lag			
	RE	FE	OLS (cluster)	AB	RE	FE	OLS (cluster)	AB
ZScore (previous week)	0.861***	0.849***	0.861***	0.852***	0.859***	0.841***	0.859***	0.835***
	(0.0157)	(0.0163)	(0.0386)	(0.0166)	(0.0151)	(0.0159)	(0.0313)	(0.0159)
OSI (2 weeks lag)	0.0111	0.0118	0.0111	−0.00819				
	(0.0131)	(0.0136)	(0.0177)	(0.0156)				
OSI (7 weeks lag)					−0.0452***	−0.0532***	−0.0452***	−0.0646***
					(0.0126)	(0.0131)	(0.0119)	(0.0137)
_cons	1.400**	1.475**	1.400**	2.375***	4.266***	4.809***	4.266***	5.380***
	(0.634)	(0.655)	(0.616)	(0.730)	(0.670)	(0.697)	(0.776)	(0.718)
*N*	1342	1342	1342	1315	1207	1207	1207	1180
*R* ^2^		0.716	0.734			0.707	0.729	

* *p* < 0.1; ** *p* < 0.05; *** *p* < 0.01.

**Table 7.** Full controls model.

ZScore (previous week)	0.907***
	(0.0254)
OSI (4 weeks lag)	−0.0578***
	(0.0177)
Trust in national governments	−0.0134
	(0.0150)
Number of physicians (1000s)	0.0344
	(0.153)
Health spending per capita (1000s of dollars)	−0.109
	(0.127)
Number of intensity care beds	0.000130
	(0.00130)
Population density (1000s per square m)	4.346**
	(1.696)
Constant	4.372***
	(1.430)
Observations	1048
R-squared	0.784

**p* < 0.1; ** *p* < 0.05; *** *p* < 0.01.
